# Replication and partitioning of the apicoplast genome of *Toxoplasma gondii* is linked to the cell cycle and requires DNA polymerase and gyrase

**DOI:** 10.1016/j.ijpara.2020.11.004

**Published:** 2021-05

**Authors:** Érica S. Martins-Duarte, Lilach Sheiner, Sarah B. Reiff, Wanderley de Souza, Boris Striepen

**Affiliations:** a Laboratório de Quimioterapia de Protozoários Egler Chiari, Departamento de Parasitologia, Instituto de Ciências Biológicas, Universidade Federal de Minas Gerais, Belo Horizonte, MG, Brazil; b Núcleo de Biologia Estrutural e Bioimagens (CENABIO) - Instituto Nacional de Ciência e Tecnologia em Biologia Estrutural e Biomagens (INBEB), Rio de Janeiro, RJ, Brazil; c Wellcome Centre for Integrative Parasitology, University of Glasgow, 120 University Place Glasgow, United Kingdom; d Department of Cellular Biology, University of Georgia, Athens, GA, USA; e Laboratório de Ultraestrutura Celular Hertha Meyer, Instituto de Biofísica Carlos Chagas Filho, Universidade Federal do Rio de Janeiro, Rio de Janeiro, RJ, Brazil; f Department of Pathobiology, School of Veterinary Medicine, University of Pennsylvania, Philadelphia, PA. USA

**Keywords:** Toxoplasmosis, *Plasmodium*, Nucleoid, Plastid, DNA polymerase

## Abstract

Apicomplexans are the causative agents of numerous important infectious diseases including malaria and toxoplasmosis. Most of them harbour a chloroplast-like organelle called the apicoplast that is essential for the parasites’ metabolism and survival. While most apicoplast proteins are nuclear encoded, the organelle also maintains its own genome, a 35 kb circle. In this study we used *Toxoplasma gondii* to identify and characterise essential proteins involved in apicoplast genome replication and to understand how apicoplast genome segregation unfolds over time. We demonstrated that the DNA replication enzymes Prex, DNA gyrase and DNA single stranded binding protein localise to the apicoplast. We show in knockdown experiments that apicoplast DNA Gyrase A and B, and Prex are required for apicoplast genome replication and growth of the parasite. Analysis of apicoplast genome replication by structured illumination microscopy in *T. gondii* tachyzoites showed that apicoplast nucleoid division and segregation initiate at the beginning of S phase and conclude during mitosis. Thus, the replication and division of the apicoplast nucleoid is highly coordinated with nuclear genome replication and mitosis. Our observations highlight essential components of apicoplast genome maintenance and shed light on the timing of this process in the context of the overall parasite cell cycle.

## Highlights

• DNA replication enzymes Prex, DNA gyrase and DNA single stranded binding protein localise to the *Toxoplasma gondii* apicoplast.
• DNA Gyrase A and B and Prex are required for apicoplast genome replication and the growth of the parasite.
• Apicoplast nucleoid division and segregation initiate at the beginning of the S phase and conclude during mitosis.
• Replication and division of the apicoplast nucleoid is highly coordinated with nuclear genomic replication and mitosis.

## Graphical abstract

**Figure:**
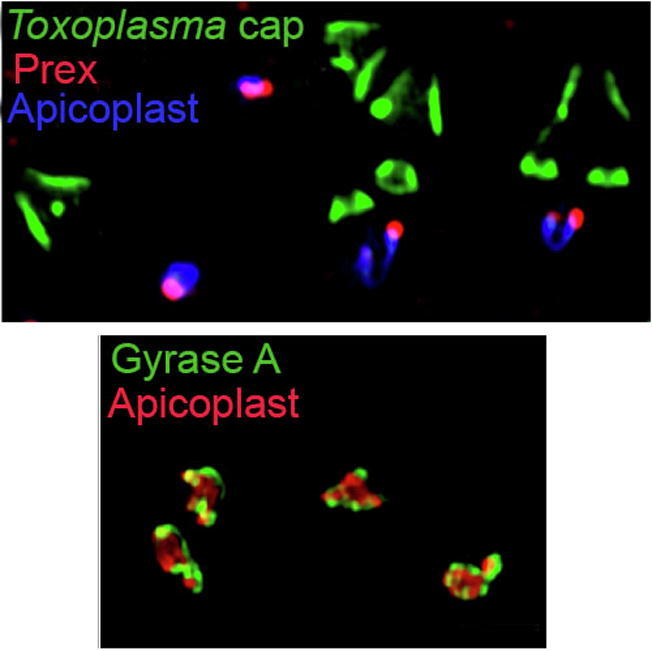

## 1 Introduction

*Toxoplasma gondii* causes toxoplasmosis, which can result in blindness, congenital defects in newborns and mortality in immuno-compromised patients (Montoya and Liesenfeld, 2004). This organism is a member of the phylum Apicomplexa and harbours several unique organelles that play important roles in enabling its intracellular parasitism. Among these, the apicoplast stands out due to its unique evolution. The apicoplast is a non-photosynthetic plastid descended from the chloroplast of a single-celled red alga that was engulfed by an apicomplexan ancestor. What makes the apicoplast an attractive target is the fact that important aspects of its biogenesis and metabolism are of prokaryotic origin, providing opportunities for the development of drugs selectively targeting the parasite (reviewed in McFadden and Yeh, 2017, Biddau and Sheiner, 2019).

Among the potential apicoplast targets is the machinery involved in the organelle’s genome replication, as it is essential for parasite survival in *T. gondii* (Fichera and Roos, 1997, Reiff et al., 2012). The apicoplast genome is a 35 kb molecule (Williamson et al., 1997) and each apicoplast in *T. gondii* has 15–25 copies of the genome (Matsuzaki et al., 2001, Reiff et al., 2012). Light microscopy observations suggest that the apicoplast genome in *T. gondii* is organised in a nucleoid-like structure (Striepen et al., 2000, Matsuzaki et al., 2001). The genome divides and segregates in a manner that is dependent on the cell cycle, prior to the division of the apicoplast and the cell (Striepen et al., 2000). However, it has been technically difficult to visualise the genome during this process of partitioning in *T. gondii*. This difficulty is due to the small size of the organellar genome and its proximity to the much larger nuclear genome, which often precludes differential identification of the nucleoid when using DNA labelling dyes such as DAPI or Hoechst.

Following the acquisition of the algal symbiont by an apicomplexan ancestor many of its chloroplast genes were laterally transferred to the nucleus and the resulting apicoplast genome encodes about 50–65 genes (Wilson et al., 1996, Cai et al., 2003, Wang et al., 2019). It is therefore likely that the proteins required for apicoplast genome replication are encoded in the nucleus and are post-translationally imported into the organelle. In support of this prediction, an apicoplast proteome analysis in *Plasmodium falciparum* identified 346 candidate proteins, including proteins with predicted roles in genome replication (Boucher et al., 2018). Genome replication is a complex multistep process and in gram-negative bacteria, such as the cyanobacteria, from which chloroplasts are derived (Reyes-Prieto et al., 2007), a detailed picture of the many proteins that organise this process has emerged. Important components of the machinery are the initiator protein DnaA for replicon assembly, DNA helicase for strand separation, DNA primase for RNA priming of both leading and lagging strands, DNA polymerase III holoenzyme for DNA synthesis, DNA polymerase I (Pol A) for DNA repair, DNA ligase to join fragments synthesised during replication, and single strand binding protein (SSB), which associates with the lagging strand and protects it from damage (Ito et al., 1998, Beattie and Reyes-Lamothe, 2015, Ohbayashi et al., 2016). DNA gyrase, a member of the topoisomerase II family, introduces negative supercoiling into double-stranded DNA during replication and transcription, thus relaxing the strands and allowing progression of the DNA replication fork (Prakash et al., 2009, Collin et al., 2011). DNA gyrase also aids in separation of the chromosome in *Escherischia coli* (Collin et al., 2011).

In chloroplasts of plants and red algae such as *Arabidopsis thaliana* and *Cyanidioschyzon merolae*, respectively, genome replication occurs without initiator protein DnaA, however, other enzymes related to genome replication, such as DNA polymerase, DNA primase, DNA helicase, SSB and Topoisomerase II, have been identified (Moriyama and Sato, 2014). In red algae plastids, the apicoplast ancestor, it was shown that cyanobacteria-type DNA polymerase III was substituted with a homolog of bacterial DNA polymerase I (DNA Polymerase A family) (Moriyama et al., 2008). Homologs of several of these proteins have been identified in apicomplexans (Mukhopadhyay et al., 2009), and are hypothesised to function in apicoplast genome replication and maintenance. A multi-functional polypeptide containing DNA primase, DNA helicase and a C-terminal domain containing both 3′5′-exonuclease and polymerase (Prex) activities was shown to be an apicoplast protein in *P. falciparum* (Seow et al., 2005, Mukhopadhyay et al., 2009), and the DNA primase domain of *P. falciparum* Prex was shown to produce RNA primers from single-stranded DNA in vitro (Lindner et al., 2011). Prex polymerase, as in red algae chloroplasts, is of bacterial origin and a member of the A-family of DNA polymerase (Mukhopadhyay et al., 2009, Kennedy et al., 2011, Schoenfeld et al., 2013). Homologs of DNA gyrase domains A and B were identified in *P. falciparum* and are targeted to the apicoplast as well (Pennisi, 2002, Raghu Ram et al., 2007). Finally, a SSB targeted to the apicoplast was also identified in *P. falciparum* and was shown to bind to single-stranded DNA in vitro (Prusty et al., 2010, Antony et al., 2012). In summary, a number of biochemical studies have shown in vitro that the apicomplexan SSB, DNA gyrase and Prex multi-domain protein show activities consistent with a role in genome replication (Seow et al., 2005, Kennedy et al., 2011, Lindner et al., 2011, Antony et al., 2012). Furthermore, attempts to ablate the Prex gene in *Plasmodium yoelii* did not succeed, providing the first indication of the essential role of Prex (Lindner et al., 2011). A genome-wide CRISPR screen in *T. gondii* aiming to identify all essential genes predicted that Prex, DNA gyrase subunits A and B, but not for SSB, were likely to be important for parasite growth (Sidik et al., 2016). However, direct evidence relating the activity of these proteins to apicoplast DNA replication in vivo is missing.

Here we constructed parasite lines expressing C-terminal endogenously tagged proteins and tetracycline-inducible mutants for Prex, DNA gyrase domains A and B, and SSB. Analysis of these mutants allowed us to provide the first known direct evidence for the essentiality of DNA gyrase and Prex for the replication of the apicoplast genome and for *T. gondii* survival. In addition, analysis of tagged parasites by structured illumination microscopy (SIM) provided new insights about the apicoplast nucleoid morphology and about the temporal relationship of apicoplast nucleoid partitioning and segregation with the cellular division cycle in *T. gondii.*

## 2 Materials and methods

### 2.1 Construction of tagged reporter parasites

*Toxoplasma gondii* ΔKu80/TATi parasites were maintained by serial passage in 25 cm^2^ culture flasks (TPP, Switzerland) of confluent human foreskin fibroblast (HFF; ATCC, USA) cells in DMEM (Gibco BRL Life Technologies, USA) supplemented with 1% FBS (Gibco Life Technologies) and 2 mM of glutamine at 37 °C and 5% CO_2_. Homologs of Prex (TGME49_261850), SSB (TGME_297940), Gyrase A (GyrA; TGME49_221330) and Gyrase B (GyrB; TGME49_297780) were identified in the *Toxoplasma* genome database (http://www.toxodb.org). To tag the genomic locus of TgPrex, TgSSB and TgGyrA with a triple hemagglutinin (HA) tag (3xHA), amplicons of the 3′ end of the genes were obtained by PCR using the primers shown in Supplementary Table S1. The amplicons were cloned via ligation-independent cloning (LIC) (Huynh and Carruthers, 2009) into the pLIC-HA-CAT vector. Parasites were transfected with plasmids and selected with chloramphenicol as previously described (Jacot et al., 2014).

Parasites endogenously expressing HA-tagged Prex (Prex-HA) and a stable extra copy of myc-tagged SSB were constructed by amplification of SSB coding sequence (cDNA) by PCR, cloned into the pDT7S4-myc plasmid and transfected into parasites expressing the HA-tagged polymerase. Modified parasites were selected with pyrimethamine, cloned into 96-well plates and then screened with primers shown in Supplementary Table S1. Parasites endogenously expressing HA-tagged GyrA (GyrA-HA) and a transient extra copy of myc-tagged GyrB were also constructed by amplification of GyrB cDNA, insertion into a pDT7S4-myc plasmid and transfection into parasites expressing GyrA-HA.

### 2.2 Conditional knockdown mutants

Fosmids containing the locus of the genes for TgPrex (RHfos25C03), TgGyrA (RHfos04P14) and SSB (RHfos20H03) were modified to insert or replace the endogenous promoter with a tetracycline-regulatable promoter, tetO7sag4 (T7S4), using the recombineering procedure as previously described (Francia et al., 2012, Vinayak et al., 2014, Sheiner et al., 2015). The gentamicin-DHFR-T7S4 cassette (Francia et al., 2012) was used as a template to amplify the cassette with 50 bp homology flanks using primers detailed in Supplementary Table S1. Modified fosmids (10–20 µg) were transfected into the respective tagged lines of ΔKu80/TATi parasites (Sheiner et al., 2011). For GyrB, plasmids for promoter insertion were constructed as previously described (Sheiner et al., 2011). Briefly, the 5′ end of the target gene was amplified by PCR from RH strain of *T. gondii* genomic DNA. The resulting amplicon was then cloned into the pDT7S4-HA plasmid between *Bgl*II and *Avr*II (enzymes acquired from New England Biolabs, USA) restriction sites. Modified parasites were selected with pyrimethamine, cloned into 96-well plates (TPP) and then screened for promoter replacement and 5′ and 3′ integration with primers shown in Supplementary Table S1.

### 2.3 Plaque assays

Confluent 25 cm^2^ flasks of HFFs were infected with 1000 parasites of the mutant or the ΔKu80/TATi parental strain and treated with 0.8 µM of anhydrotetracycline (ATc; Sigma-Aldrich Co, St. Louis, MO, USA) for 7–8 days. Flasks were then fixed with 100% ethanol and stained with crystal violet.

### 2.4 Quantitative PCR (qPCR) assay

*Toxoplasma gondii* tachyzoites were collected after egress and host cell lysis from infected HFF cultures, and DNA was purified with a DNeasy blood and tissue kit (Qiagen, USA). Quantitative PCR (qPCR) was performed using the primers and PCR protocol described previously (Wu et al., 2009, Reiff et al., 2012). Amplicons of the *UPRT* locus and the apicoplast genome were amplified using GoTaq polymerase master mix (Promega, USA) using primers and PCR programs described previously (Wu et al., 2009). PCR products were then cloned into the pCR2.1-TOPO vector (Invitrogen, Thermo Fisher Scientific, USA) to create standards. A standard curve was created for each qPCR based on serial dilutions (10^1^–10^7^) of apicoplast and nuclear genome standards. Each reaction mixture was supplied with 10 μL of iQ SYBR green supermix (Bio-Rad, USA), 1 μL of 10 μM primers, 50 ng of template DNA extracted from *T. gondii* and water totaling a final volume of 20 μL. All experiments were performed in triplicate on a Bio-Rad iQ5 real-time PCR detection system. Results were analysed using Bio-Rad iQ5 software. To determine the apicoplast genome copy number per cell, the average number of apicoplast genomes was divided by the average number of nuclear genomes.

### 2.5 Structured illumination microscopy (SIM)

Confluent HFF coverslip cultures were infected with *T. gondii* tachyzoites and, at the times indicated in the experiments (see section 3), fixed with 4% freshly prepared formaldehyde for 30 min, washed with 100 mM NH_4_Cl for 20 min to decrease background, permeabilized with 0.5% Triton X-100 for 20 min and incubated for 1 h with a blocking solution of 3% BSA in PBS. Coverslips were then incubated for 1 h with primary antibodies against a luminal apicoplast protein (1:2000 rabbit anti-Cpn60; Agrawal et al., 2009), the inner membrane complex subcompartment protein 1 (ISP1) for the inner membrane cap (1:1000 mouse anti-ISP1/mAb7E8; Beck et al, 2010), the centrosome (1:500 rabbit anti-Centrin 1; kindly provided by Dr Iain Cheeseman (Massachusetts Institute of Technology, USA); Lorestani et al., 2012), rat anti-HA (1:200; clone 3F10, Roche Applied Science, USA) and mouse anti-myc (1:200; Sigma-Aldrich). Secondary antibodies used were Alexa 350, Alexa 488 or Alexa 546 goat anti-mouse or anti-rabbit and Alexa 488 goat anti-rat (Molecular Probes, USA). Nucleus and apicoplast genomes were stained with 5 µg/ml of DAPI (Sigma-Aldrich) solution. Coverslips were mounted with Fluoro-gel (Electron Microscopy Sciences, USA) and observed on a Zeiss Elyra SR-SIM microscope (Zeiss, Germany) with a 100x oil immersion objective. Images were acquired with ZEN software (Zeiss) with a SIM analysis module and analysed with ImageJ.

### 2.6 Cryo-immuno electron microscopy

For HA-tagged GyrA, SSB or Prex, free tachyzoites were recovered following egress from HFF cultures and fixed for 2 h with 4% freshly prepared formaldehyde and 0.05% glutaraldehyde in PBS. Parasites were washed with PBS and the parasite pellets were embedded in a 10% gelatin solution and infiltrated by immersing in 2.3 M sucrose at 4 °C overnight. The infiltrated parasite pellets were trimmed, mounted and flash frozen by immersion in liquid nitrogen. Ultrathin sections (70–80 nm) were obtained using a cryo-ultramicrotome (Leica EM FC6), collected with 2.3 M sucrose and mounted on copper grids covered with a formvar film. The ultrathin sections were rehydrated in 2% gelatin, blocked with 3% BSA (Sigma-Aldrich) in PBS and labelled with rat anti-HA antibody for 1 h followed by 10 nm gold conjugated goat anti-rat IgG (Sigma-Aldrich). Sections were stained with 0.5% uranyl acetate in 2% methylcellulose and observed on a Zeiss 900 transmission electron microscope.

### 2.7 Western blot analysis

Harvested tachyzoites were resuspended in reducing sample buffer NuPage LDS 1× (Invitrogen, Thermo Fisher Scientific) with 2% β-mercaptoethanol. Samples were boiled for 5 min at 95 °C. Lysates were then run by SDS-PAGE on Tris-Glycine 6–12% gradient gels (Bio-Rad) before transfer to 0.2 μm nitrocellulose membranes and subsequent antibody labelling. Rat anti-HA (Roche Applied Science) was used at 1:100 and mouse anti-α-Tubulin (Sigma-Aldrich) was used at 1:10,000. Horseradish peroxidase (HRP)-conjugated goat anti-rat and goat anti-mouse (Bio-Rad) secondary antibodies were used at 1:5000.

### 2.8 GyrB transcript analysis

GyrB mutants were grown for 3 days in HFF cells in the presence or absence of ATc. After that, parasites were harvested, RNA isolated (RNeasy kit Qiagen) and cDNA synthesised (SuperScript III reverse transcriptase kit, Invitrogen, Thermo Fisher Scientific). The presence of GyrB transcript was assessed by PCR of 1 μg of cDNA using primers annealing to GyrB cDNA listed in Supplementary Table S1. Primers annealing to Sag1 (Supplementary Table S1) were used in loading controls.

## 3 Results

### 3.1 GyrA and B, SSB and Prex are targeted to the apicoplast, but show different patterns of organellar distribution

**Fig. 1:**
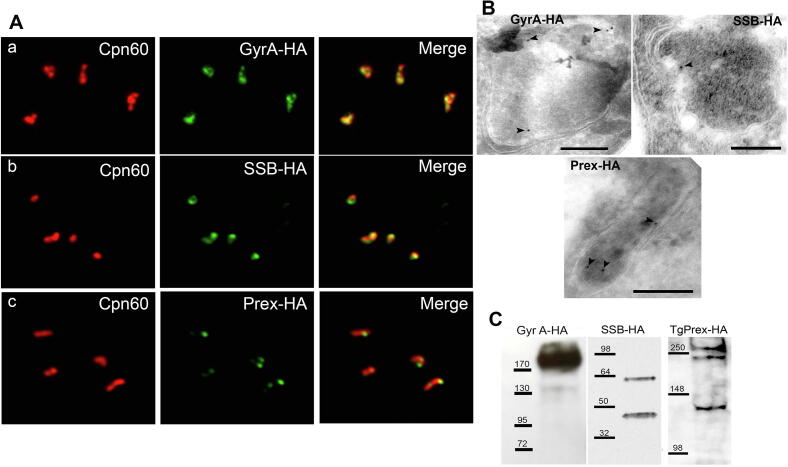
Gyrase A (GyrA) and B (GyrB), single strand binding protein (SSB) and Prex (multi-functional polypeptide containing DNA primase, DNA helicase and DNA polymerase) are targeted to the *Toxoplasma gondii* apicoplast. (A) Fluorescence image of (a) hemagglutinin (HA)-tagged GyrA (GyrA-HA), (b) SSB-HA and (c) Prex-HA parasites labelled with anti-Cpn60 (red) and anti-HA (green). Images were acquired on a Deltavision II Olympus inverted microscope. (B) Cryo-immuno electron microscopy of parasites expressing endogenous HA tagged-DNA polymerase, SSB and GyrA confirmed apicoplast localization. Bars = 200 nm. (C) Western blot analysis of parasites expressing endogenously HA-tagged GyrA, SSB and Prex. Proteins showed the predicted size and higher mobility bands corresponding to mature proteins after transit peptide processing.

To assess the localization of putative apicoplast genome replication components in *T. gondii*, we constructed strains that express endogenously HA-tagged Prex, SSB and DNA Gyrase A (see section 2 for details). We note that we were unable to isolate parasites with an endogenously tagged Gyrase B gene, suggesting that such a modification may be detrimental to parasite growth. We conducted immunofluorescence labelling of the tagged parasites using an antibody to the apicoplast chaperone Cpn60 and anti-HA (Fig. 1A), and demonstrated all three proteins are targeted to the apicoplast. Cryo-electron microscopy also demonstrated that all three proteins are localised at the apicoplast (Fig. 1B; note the multiple membranes that delineate the organelle in cryo-electron microscopy). More details of SSB by cryo-electron microscopy are provided in Supplementary Fig. S1. Nuclear encoded apicoplast proteins are typically proteolytically processed as they traffic to the organelle. We conducted Western blot analysis (Fig. 1C) and found the apparent molecular mass of SSB-HA (60 kDa), Prex (280 kDa) and GyrA (176 kDa) to be consistent with that predicted by their current gene model in ToxoDB.org (TGME49_297940, TGME49_261850, TGME49_221330, respectively). The western blot also showed higher mobility bands likely corresponding to the mature proteins after transit peptide processing upon apicoplast import. Prex showed a third band (approximately 130 kDa). This may indicate further processing and correspond to the c-terminal Exonuclease/DNA Polymerase I domain of Prex as previously shown for *P. falciparum* (Seow et al., 2005).

**Fig. 2:**
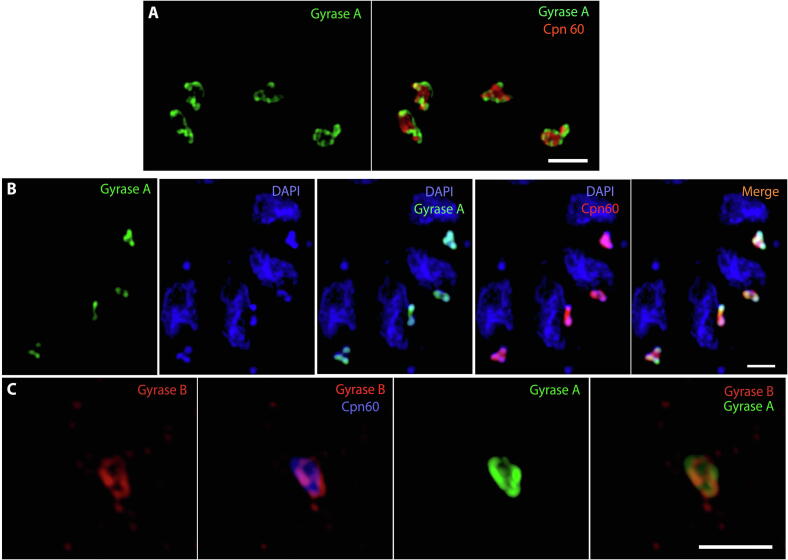
Super-resolution Structured Illumination Microscopy analysis of *Toxoplasma gondii* parasites expressing endogenous hemagglutinin (HA)-tagged GyrA and transient myc-tagged GyrB (A) Parasites labelled with anti-HA for GyrA (green) and anti-Cpn60 for the apicoplast lumen (red) showed that GyrA is distributed along the periphery of the apicoplast. (B) Parasites labelled with anti-HA for GyrA (green), anti-Cpn60 for the apicoplast lumen (red) and DAPI for nuclear and apicoplast DNAs (blue), showed that GyrA localises close to apicoplast DNA. (C) Analysis of GyrA-HA (green) parasites transiently expressing an ectopic copy of myc-tagged GyrB (red) showed that both proteins co-localise, confirming the peripheral distribution of DNA gyrase in the apicoplast (blue). Bars = 1 µm.

Cryo-immuno electron microscopy of GyrA-HA showed peripheral localization, close to the innermost membrane of the apicoplast (arrowheads in Fig. 1B). To substantiate this observation we used SIM. Fig. 2A shows the lumen of the apicoplast in red using anti-Cpn60 for the apicoplast and GyrA in green. We note that GyrA-HA (Fig. 2A) is distributed along the periphery of the apicoplast. To analyse GyrA localization in relation to apicoplast DNA, parasites were labelled with anti-Cpn60 (red), anti-HA for GyrA (green) and DAPI (blue) (Fig. 2B). Fluorescence intensity distribution analysis of merged images of apicoplast DNA and GyrA showed that the fluorescence signals of apicoplast DNA and GyrA partially overlap (Fig. 2B, Supplementary Fig. S2). However, the pattern of distribution of GyrA is distinct from Cpn60 protein (Fig. 2A and Supplementary Fig. S2), and a higher intensity signal of GyrA is observed when in co-localization with apicoplast DNA, providing evidence that GyrA concentrates proximal to the apicoplast genome. To confirm the peripheral localization of DNA gyrase, GyrA-HA parasites transiently expressing an ectopic copy of myc-tagged GyrB (stable expression of tagged-GyrB is not tolerated by *T. gondii*) was analysed by SIM (Fig. 2C). In agreement with what was seen with GyrA (green), GyrB (red) also showed peripheral distribution in the apicoplast (blue). Fluorescence intensity signals of GyrA and GyrB showed a similar pattern in the distribution of peak intensities, confirming their co-localization (Supplementary Fig. S3).

**Fig. 3:**
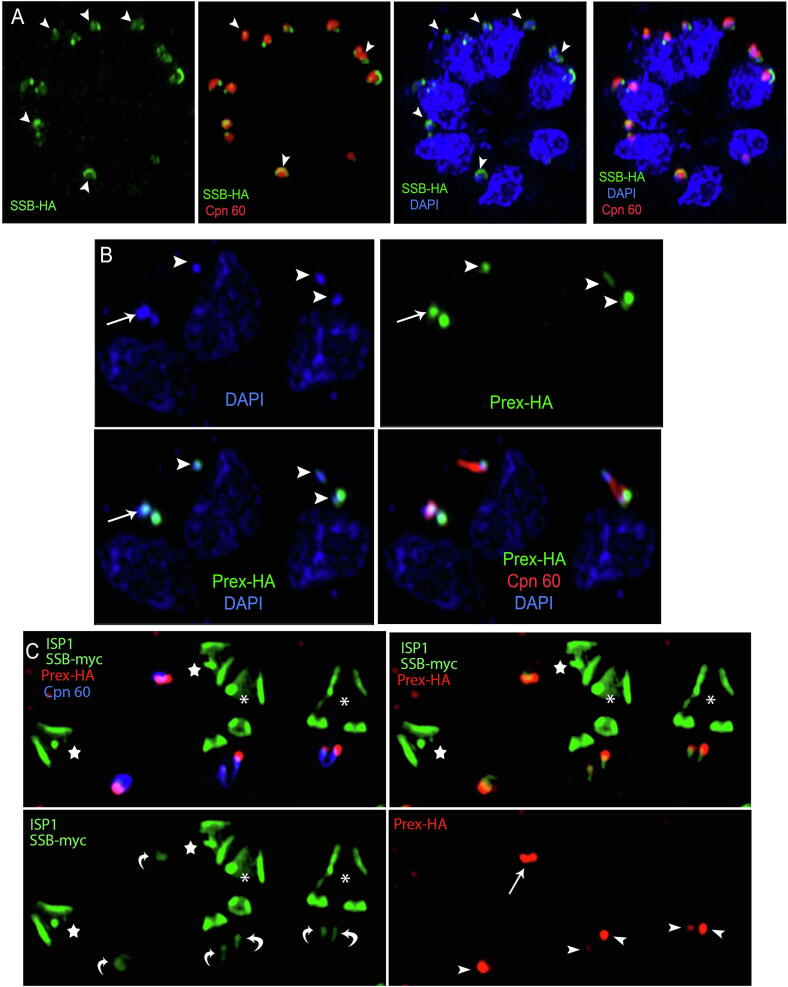
Super-resolution Structured Illumination Microscopy analysis of *Toxoplasma gondii* parasites expressing endogenous hemagglutinin (HA)-tagged SSB (SSB-HA) and Prex (Prex-HA). Analysis after labelling with with anti-HA for SSB-HA (A) or Prex-HA (B) (green), anti-Cpn60 for apicoplast lumen (red) and DAPI for nuclear and apicoplast DNA (blue) showed that: (A) SSB-HA accumulates around the margins of the apicoplast genomes (arrowheads) and (B) Prex concentrates into distinct foci that co-localise with apicoplast DNA, matching perfectly the number and position to the nucleoid(s) in each organelle. It is possible to discern three different types of nucleoid: a single spherule (arrowhead), two spherules (arrowheads) per apicoplast and a single nucleoid with dumbbell shape (arrow). (C) Analysis of Prex-HA (red) parasites expressing ectopic myc-tagged SSB (green; anti-myc antibody) after labelling with anti-Cpn60 (blue) and with an antibody against the protein ISP1 (green) showed that Prex (arrowheads) and SSB (curved arrows) are closely localised in both dividing (asterisks) and non-dividing (stars) parasites.

In another set of experiments we analysed SSB-HA (green in Fig. 3A and Supplementary Fig. S4) and Prex-HA (green in Fig. 3B and C) distribution and localization in relation to the apicoplast genome, while co-labelling parasites with anti-Cpn60 for apicoplast lumen (red) and DAPI for genome (blue). SSB-HA appeared to accumulate around the margins of the genome, often forming cup-like structures (arrowheads in Fig. 3A). Prex staining was concentrated into distinct foci that overlapped with apicoplast DNA staining (Fig. 3B). We observed either one or two such foci per apicoplast, matching the number and position to the nucleoid(s) in each organelle (arrow and arrowheads in Fig. 3B and Supplementary Fig. S5). We noted three discernable patterns: apicoplasts containing a single small spheric nucleoid, two distinct spheric nucleoids (arrowheads), or one single nucleoid with a dumbbell shape (arrow) (Fig. 3B). Dumbbell-shaped nucleoids may represent intermediates of nucleoid segregation, consistent with previous studies with algal plastids, and *E. coli* and cyanobacteria (Kuroiwa et al., 1981, Kuroiwa et al., 2020, Madabhushi and Marians, 2009). We note that Prex consistently localises to both ends of such dumbbells (arrow in Fig. 3B and Supplementary Fig. S5).

To study the localization of Prex and SSB in dividing and non-dividing parasites, Prex-HA parasites expressing ectopic myc-tagged SSB were labelled with anti-Cpn60 and with an antibody against the protein ISP1, which localises to the apical cap and is also present in early daughter cell buds during the *T. gondii* division process (Beck et al., 2010), and thus serves as a morphological marker to reveal dividing parasites (Beck et al., 2010, Frénal et al., 2017). Prex-HA and SSB-myc are closely apposed in both dividing (asterisks) and non-dividing (stars) parasites (Fig. 3C and Supplementary Fig. S6) and, as seen above, are not evenly distributed within the apicoplast lumen (compare with Cpn60 in blue). While Prex-HA typically concentrated into one or two focal spots (arrow in Fig. 3C), SSB-myc is distributed along a thin line (curved arrow in Fig. 3C).

Taken together, these observations show that SSB, GyrA, GyrB and Prex are nuclear encoded proteins that traffic to the lumen of the apicoplasts, where they appear to localise to different regions of the organelle associated with its genome.

### 3.2 Apicoplast nucleoid segregation is temporally linked to the *T. gondii* cell cycle

**Fig. 4:**
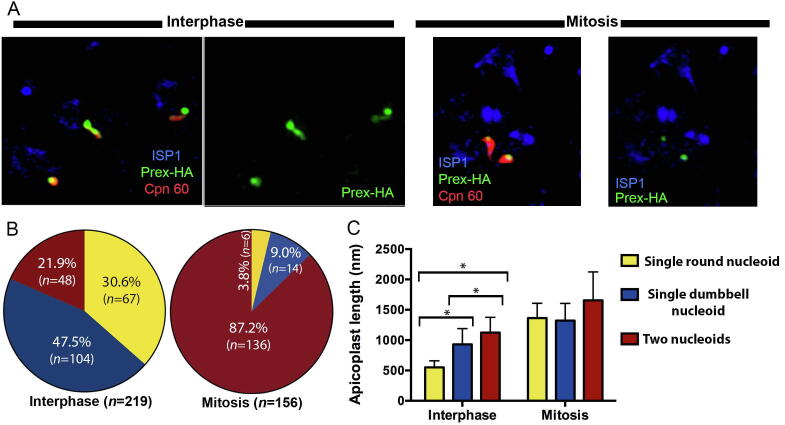
*Toxoplasma gondii* apicoplast nucleoid segregation occurs concurrently with apicoplast elongation during the cell cycle. (A) Representative image of analysed parasites after labelling with antibodies against mother and daughter cell inner membrane complex cap (anti-ISP1) showed parasite mitosis, anti-Cpn60 for apicoplast lumen and anti-hemmaglutinin (HA) for Prex-HA (multi-functional polypeptide containing DNA primase, DNA helicase and DNA polymerase). (B) Quantification of Prex number and morphology in interphase or mitotic parasites. In total 375 parasites were analysed: 219 in interphase and 156 in mitosis. (C) Analysis of apicoplast length according to nucleoid morphology of parasites quantified in Fig. 2B. Apicoplast length was measured in ImageJ software using the segmented line tool. *P < 0.05, one-way ANOVA with Tukey’s multiple comparisons test.

We know from previous studies that continuous maintenance of the apicoplast genome is required for parasite survival and that loss of the genome results in parasite demise (Fichera and Roos, 1997, Reiff et al., 2012). However, exactly how the nucleoid is partitioned during organelle division has been difficult to observe. Here we took advantage of our tagged line to observe the temporal relationship between nucleoid replication and segregation, and the nuclear cell cycle. Following the observation that Prex foci and apicoplast nucleoids show tight correlation in number and morphology, we conducted further experiments that used Prex spots as a proxy for the nucleoid. In the first set of experiments, we used detection of Prex together with antibodies against the apicoplast luminal marker Cpn60 and ISP1 (Fig. 4). The presence of two daughter cell buds highlighted by ISP1 is indicative that the parasite is in the M phase (Francia and Striepen, 2014). Quantitative analysis of Prex labelling showed robust differences between interphase (G1 and S phases) and M phase parasites (Fig. 4A). In interphase parasites, 78.1% of apicoplasts contained just a single nucleoid, while 21.9 % contained two, whereas in M phase parasites 87.2% of apicoplasts contained two nucleoids (Fig. 4B). In interphase parasites, among the apicoplasts with a single nucleoid, 30.6% contained a single round nucleoid and 47.5% contained a single dumbbell nucleoid. Analysis of the relationship between nucleoid number/morphology (Fig. 4C) and apicoplast length showed that in interphase parasites the apicoplasts containing a single dumbbell nucleoid or two nucleoids are significantly longer (*P* < 0.05) than the apicoplasts containing a single round nucleoid (Fig. 4C). Similarly, apicoplasts containing two nucleoids are significantly longer than apicoplasts containing single dumbbell nucleoids. These results suggest that nucleoid partition occurs concurrently to apicoplast elongation, but apicoplast elongation is not dependent on nucleoid partition.

**Fig. 5:**
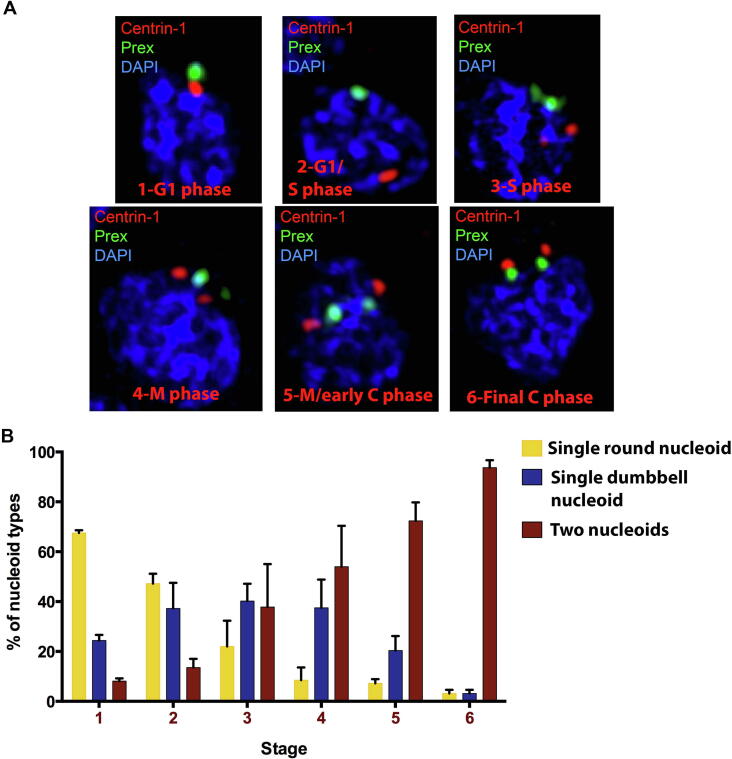
Nucleoid segregation initiates at the beginning of nuclear DNA synthesis (S) and early mitosis (M) phases in *Toxoplasma gondii*. Parasites were labelled with DAPI for nuclear and apicoplast DNA (blue), anti-Centrin 1 for centrosome (red) and anti-hemagglutinin (HA) for Prex (DNA polymerase) (green). (A) Centrosome localization and number depict the different cell division cycle stages of *T. gondii*: (1) a single centrosome apical to the nucleus during G1phase. (2) The single centrosome migrates to the nucleus at the beginning of the S phase. (3) Centrosome division and paired migration back to the apical end (S phase). (4) The centrosomes coordinate the mitotic spindle and daughter bud formation is initiated (M phase). (5) The centrosomes associate with the apicoplast ends and the final extension of the organelle (M/early cytokinesis phase). (6) Apicoplasts and centrosomes are inherited by each daughter cell following cytokinesis (final cytokinesis phase). (B) Quantification of the number and morphology of Prex along the cell division phases 1–6. Mean ± S.D. of two independent groups (numbers of cells evaluated were 491 for experiment 1 and 343 for experiment 2). Statistical analysis was performed by two-way ANOVA and is shown in Supplementary Tables S2 and S3.

Next, to determine the exact stage in which segregation of the apicoplast nucleoid occurs, we analysed nucleoid morphology in the context of the centrosome division cycle by using antibodies to centrin-1 (Fig. 5). We used a previously established staging scheme to define progression through the *T. gondii* cell cycle (Striepen et al., 2000, White et al., 2005, Hartmann et al., 2006, Hu, 2008, Lévêque et al., 2015, Suvorova et al., 2015). We scored six stages, each characterised by the specific number and position of the centrosomes with respect to the parasite nucleus (Fig. 5A): (1) a single centrosome apical of the nucleus during G1; (2) the single centrosome migrates to the nucleus at the beginning of S phase; (3) centrosome division and paired migration back to the apical end (S phase); (4) the centrosomes coordinate the mitotic spindle and daughter bud formation initiates (M phase); (5) the centrosomes associate with the apicoplast ends (here shown by the lateral localization of Prex between the centrosomes) and the final extension of the organelle (M phase and early cytokinesis); (6) apicoplasts and centrosomes are inherited by each daughter following cytokinesis. Analysis of the HA-tagged Prex alongside the centrosome across these stages showed that in Stage 1 (G1 phase) most apicoplasts contain just a single round nucleoid (67.5%; *P* < 0.05 Supplementary Table S2), which then show a significant gradual decrease along subsequent stages 2–5 (47.2% and 22%, respectively; Fig. 5B and Supplementary Table S3). Similarly, a significant gradual increase in the proportion of apicoplasts with two nucleoids was seen between stages 2–5 (Fig. 5B) and at Stage 6 (end of cytokinesis phase) most apicoplasts (93.7%) contained two nucleoids. Interestingly, the proportion of apicoplasts with a single dumbbell nucleoid remained nearly constant during stages 2 to 5 (Supplementary Table S3). This suggests that at the same time/stage that new dumbbells are being formed in some cells, nucleoids in other cells have completed division. Thus, nucleoid partitioning can happen during a range of stages. Statistical analyses of nucleoids within and between stages are available in Supplementary Tables S2 and S3. In summary, these results show that apicoplast nucleoid partitioning initiates at the beginning of the S phase (Stage 2), prior to centrosome division and mitotic spindle coordination, as previously suggested (Striepen et al., 2000), and proceeds until initiation of daughter cell formation and the beginning of cytokinesis (Stages 4–5).

### 3.3 DNA gyrase and Prex are essential for apicoplast genome replication and parasite viability

**Fig. 6:**
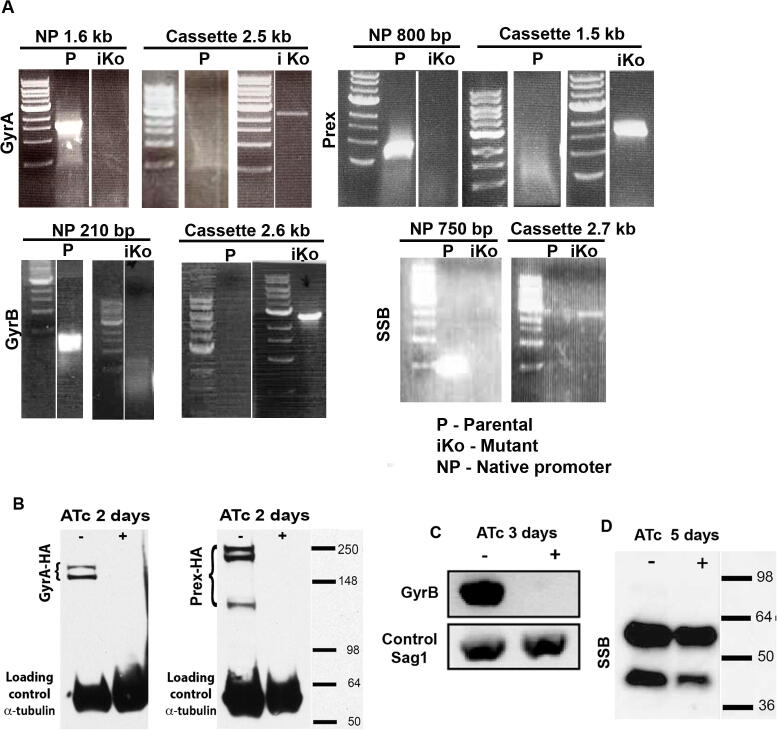
Validation of the inducible knockouts. (A) Validation of the promoter in the *Toxoplasma gondii* parental line (primers 27 and 28 for Gyrase A (GyrA); 30 and 31 for Prex (multi-functional polypeptide containing DNA primase, DNA helicase and DNA polymerase); 19 and 20 for GyrB; 25 and 26 for single strand binding protein (SSB) and promoter replacement or integration in the TgPrex (primers 29 and 33), T*g*GyrA (primers 28 and 32), TgGyrB (primers 26 and 32) and TgSSB (primers 25 and 32) loci via PCR analysis; primers are shown in Supplementary Table S1. (B) Protein immunoblot analysis of whole cell lysate from hemagglutinin (HA)-tagged Prex (Prex-HA) and GyrA (GyrA-HA) after growth in the absence (−) or presence (+) of ATc [anhydrotetracycline] for 2 days. (C) Transcript levels of GyrB amplified by PCR from cDNA pools of parasites grown in the absence (−) or presence (+) of ATc for 3 days. (D) Protein immunoblot analysis of whole cell lysate from SSB-HA after growth in the absence (−) or presence (+) of ATc for 5 days. P, parental; NP, native promoter; iKo, inducible knockout.

We next examined whether the *T. gondii* Prex, DNA gyrase A and B subunits, and SSB are essential for the replication and inheritance of the apicoplast genome. We constructed Tet-regulatable mutants using fosmid recombination (Vinayak et al, 2014), to replace (or insert into) the respective native promoters with a Tet-regulatable promoter (Fig. 6A). PCR analysis of obtained mutants and the parental line showed the amplification of expected band sizes (GyrA 1.6 kb, Prex 800 bp, GyrB 210 bp, SSB 750 bp) for the native promoter region only in parental line, but not in mutants. The Tet-regulatable promoter was only amplified in mutants (GyrA 2.5 kb, Prex 1.5 kb, GyrB 2.6 kb, SSB 2.7 kb) but not in the parental line (Fig. 6A). For Prex, GyrA and SSB these modifications were generated using the respective HA-tagged lines as the parental background. This allowed us to monitor down-regulation of the target gene in response to addition of ATc by western blot (Fig. 6B and D). After 2 days of ATc treatment, Prex and GyrA mutants showed no detectable protein (Fig. 6B). Due to the absence of a tag, we were unable to measure protein levels directly after downregulation of GyrB, and thus we examined transcript levels. GyrB could be amplified by PCR from cDNA pools of parasites untreated with ATc, but not from parasites treated for 3 days with ATc (Fig. 6C). Amplification of a control cDNA remained at similar levels to non-induced parasites (Fig. 6C). In contrast, growth in the presence of ATc did not result in the loss of SSB in the SSB promoter replacement mutant (Fig. 6D).

**Fig. 7:**
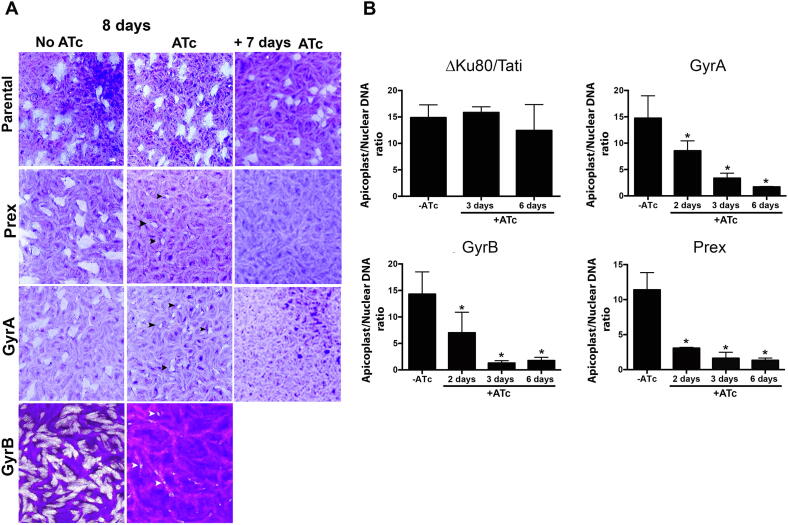
Analysis of Prex (multi-functional polypeptide containing DNA primase, DNA helicase and DNA polymerase), Gyrase A (GyrA) and GyrB *Toxoplasma gondii* mutants after knockdown with ATc (anhydrotetracycline). (A) Plaque assays performed with the parental line (RHΔku80/TATi), hemagglutinin (HA)-tagged Prex (Prex-HA), GyrA (GyrA-HA) and GyrB parasites grown for 8 days in the absence (−) or presence (+) of ATc. Additional 7 day plaque assays were performed with recovered parasites from the parental line, Prex-HA and GyrA-HA. Black and white arrowheads indicate plaques. (B) Quantification of the apicoplast genome in the parental line, Prex-HA, GyrA-HA and GyrB grown for 2, 3 and 6 days in the presence of ATc. Mean ± S.D. of three independent experiments. Statistical analysis performed using one-way ANOVA with Dunnett’s multiple comparisons test. *P < 0.05.

The ability of the Prex, GyrA and GyrB mutants to form plaques in a host cell monolayer (plaque assay) was used to assess the growth and viability of parasites in the presence or absence of ATc. For all three mutants we observed a strong growth defect in the presence of ATc that was not seen for the parental lines (Fig. 7A), but noted the formation of small plaques (arrowheads). This might indicate that loss of these proteins merely slows parasite growth, or alternatively that there is a delay in the development of the phenotype that, once manifested, blocks further growth. To distinguish these possibilities, we performed the plaque assay following growth of GyrA and Prex with ATc for 7 days (Fig. 7A); under these conditions no plaques were formed. We thus concluded that DNA gyrase and Prex are required for continued growth of *T. gondii* in culture. We next sought to evaluate the impact of loss of these proteins on apicoplast genome maintenance. We measured the copy number of the apicoplast genome relative to the nuclear genome by qPCR in parasites grown in the presence and absence of ATc for 6 days (Fig. 7B). Treatment of the parental line with ATc for 6 days did not have a significant effect on the copy number of the apicoplast genome (Fig. 7B). In contrast, knockdown in all three mutants resulted in marked and progressive reduction in apicoplast genome copy numbers (Fig. 7B). This reduction was significant (*P* < 0.05) within 2 days of ATc-mediated repression in all three strains.

We also measured the impact of loss of Prex, GyrA and GyrB on overall apicoplast maintenance and inheritance by counting the number of organelles per parasite (Supplementary Fig. S7). While in the parental line ATc treatment had no effect on apicoplast numbers (Supplementary Fig. S7), the knockdown of GyrA, GyrB and Prex resulted in plastid loss (Supplementary Fig. S7). Apicoplast analysis in the GyrA mutant by SIM after treatment with ATc for 2 days showed that some parasites harboured small sized organelles compared with non-induced GyrA parasites (arrowheads in Supplementary Fig. S8). Overall, we confirm that Prex, GyrA and GyrB are important for apicoplast genome maintenance, apicoplast retention and consequently for parasite growth.

## 4 Discussion

All plastids studied to date utilise the machinery they inherited from their prokaryotic ancestors to perform DNA replication. Here we provide evidence that the same is true for the apicoplast. The endogenously tagged *Toxoplasma* homologs of Gyrase A and B, SSB and Prex confirmed their expected residence in the apicoplast (Fig. 1, Fig. 2, Fig. 3). Further, the close localization of three of these proteins with the apicoplast nucleoid is in line with their expected role in the replication of its genome. While Prex tightly co-localises with the nucleoid, the other three proteins showed different distributions within the apicoplast. We hypothesise that these differences may be related to other functions that these proteins perform in addition to their role in apicoplast genome replication. For example, DNA gyrase is known to act by introducing negative DNA supercoils, allowing the continued progress of the replication machinery (Collin et al., 2011). In addition, considering the lack of a topoisomerase IV in the apicoplast (Lin et al., 2015), DNA gyrase was proposed to take part in decatenation of intertwined DNA, which is essential for chromosome segregation after the completion of DNA replication (Nagano et al., 2014, Lin et al., 2015). DNA gyrase also was shown to have an important role in genome segregation and decatenation in plant chloroplasts (Steck and Drlica, 1984, Cho et al., 2004). This second role may explain the peripheral localization of the Gyrase subunits (Fig. 2). Similarly, SSB acts by binding to single stranded DNA not only during DNA replication, but also during repair and recombination, protecting the DNA against nuclease attacks and against the formation of secondary structures (Kur et al., 2005). The inability to tag GyrB C-terminally in the endogenous locus may be linked to the fact that a C-terminal tag is detrimental to the function of homologs of this protein in other species, as this region is responsible for the interactions with Gyrase A and with the DNA (Collin et al., 2011, Chan et al., 2015).

SIM analysis of DAPI-stained apicoplast DNA and HA-tagged Prex confirmed that similar to other plastids, the apicoplast genome is organised in a single spheric nucleoid-like structure (Fig. 2, Fig. 3) (Kabeya et al., 2010, Kuroiwa et al., 2020). We further showed that during apicoplast division the nucleoid assumes a dumbbell shape prior to the two-nucleoid stage, suggesting it is in the process of partitioning. Similar morphology was reported for red and green algal chloroplasts and prokaryotic nucleoids during the partitioning process (Suzuki et al., 1981, Ehara et al., 1990, Miyamura et al., 1990, Woldringh et al., 1994, Popławski and Bernander, 1997, Imoto et al., 2010).

Analysis of parasites expressing the endogenously HA-tagged Prex reveal that partitioning of the apicoplast genome occurs in synchrony with cell division, although it is uncoupled from nuclear genome replication (S phase) and division (M and cytokinesis phases). Nucleoid partitioning initiates early in the S phase (Fig. 5B), suggesting that apicoplast genome replication occurs during the G1 phase, such as was seen for the red alga *C. merolae* and the green alga *Chlamydomonas reinhardtii* (Suzuki et al., 1994, Kabeya and Miyagishima, 2013, Kuroiwa et al., 2020). In line with this finding, our analysis of the Prex signal throughout the centrosome cycle showed an increase in the numbers of single dumbbell nucleoids and of two nucleoids at the beginning of the *T. gondii* S phase (Fig. 5). Further, during the course of S phase and until the beginning of M phase, a significant decrease in the number of single round nucleoids with a concurrent significant progressive increase in the number of two nucleoids were observed (Fig. 5B). Finally, when the parasites reached the M and cytokinesis phases (Figs. 4B and 5B), apicoplasts containing divided nucleoids were the most commonly seen forms (Figs. 3B and 4B). Additionally, apicoplasts containing a single dumbbell nucleoid or two nucleoids were progressively longer than apicoplasts containing a single round nucleoid (Fig. 4C). Taken together, these observations show that apicoplast nucleoid segregation and division coincide with the apicoplast elongation process in the S phase and early mitosis during the *T. gondii* cell cycle (Nishi et al., 2008). Thus, nucleoid division and segregation occur prior to nuclear karyokinesis, and before the segregation of the apicoplast and its division into the newly forming daughter cells at the beginning of cytokinesis (Figs. 4B and 5B) during *T. gondii* endodyogeny (Striepen et al., 2000, Nishi et al., 2008). Similar findings were made for *C. reinhardtii* and *C. merolae*, which are single chloroplast-containing algae. In these organisms, the chloroplast nucleoid also divides during the S phase prior to mitosis (Grant et al., 1978, Suzuki et al., 1994). The concurrent nucleoid partitioning during elongation of the apicoplast during parasite division could guarantee that the divided nucleoids localise to the poles of the elongated apicoplast, ensuring the correct inheritance of the nucleoids by each divided apicoplast. Such is also the case in bacteria (Toro and Shapiro, 2010). However, what regulates apicoplast gene replication is not yet known. A study with the green alga *C. reinhardtii* showed that the chloroplast genome replication is regulated by the redox state sensed by the nucleoids and that the disulfide bonds in nucleoid-associated proteins are involved in this regulatory activity (Kabeya and Miyagishima, 2013). Recently it was demonstrated in *T. gondii* that the redox balance is also important for regulating apicoplast functions (Biddau et al, 2018). Conditional depletion in *T. gondii* of two apicoplast thioredoxins, which are enzymes that mediate disulphide-dithiol dynamics in target proteins in response to compartmental redox states, drastically reduced apicoplast gene expression and protein trafficking. Further, thioredoxin depletion also caused a reduction in the apicoplast genome copy number (Biddau et al., 2018). These results suggested that, as was seen for *C. reinhardtii*, the apicoplast redox state could also be involved in regulating the apicoplast genome replication and future studies in this field may contribute to solving this open question.

The knockdown of DNA gyrase subunits A and B and Prex (Fig. 7A) impaired *T. gondii* proliferation, confirming that they are important for parasite fitness. Moreover, 1 week of pre-treatment with ATc abolished parasite proliferation (Fig. 7A) in line with the expected essential nature of the apicoplast genome for parasite survival. Comparably, in some plant mutants for chloroplast GyrB and DNA polymerase a small population could still grow, even if aberrantly (Wall et al., 2004, Morley and Nielsen, 2016, Udy et al., 2012). In the case of SSB, despite the correct integration of the inducible promoter, expression was not reduced with ATc (Fig. 6C), preventing assessment of its role in apicoplast genome replication and parasite survival. However, the recent genome-wide CRISPR/CAS9 screen for genetic contribution to fitness of *T. gondii* in culture suggests that SSB is not essential for tachyzoite survival in culture (Sidik et al., 2016).

The effect on parasite viability seen in the conditional mutants following the decrease in the apicoplast genome copy number and apicoplast loss (Fig. 7B and Supplementary Fig. S7) is in line with the essential role of the apicoplast genome for the parasite’s survival. Previous work had shown that treatment of *T. gondii* and *P. falciparum* with the antibiotics ciprofloxacin and novobiocin, known inhibitors of prokaryote gyrase A and B domains, respectively, decreased apicoplast genome replication and affected parasite viability and morphology (Fichera and Roos, 1997, Weissig et al., 1997, Raghu Ram et al., 2007, Martins-Duarte et al., 2015). This is consistent with the results of the current study.

In the plant *Zea mays,* mutants of the chloroplast DNA polymerase showed reduced chloroplast genome copy numbers which result in a decrease in the chloroplast-encoded transcripts and proteins (Udy et al., 2012). In the apicoplast genome most of the its genes are involved in its own gene expression (for example, tRNAs, RNA polymerase subunits and ribosomal proteins) (Gardner et al., 1991, Gardner et al., 1994, Wilson et al., 1996), however two protein-coding genes with defined functions are present: SufB and ClpC (Wilson et al., 1996). The presence of both genes is possibly the reason for genome retention and its necessity in apicomplexan plastids (Janouskovec et al., 2015). Apicoplast SufB is related to bacterial sufB (Ellis et al., 2001) and might be involved in apicoplast iron-sulphur cluster synthesis, while ClpC is a subunit of ATP-dependent Clp protease, which is possibly involved in importing nuclear encoded apicoplast proteins into the organelle (El Bakkouri et al., 2010, Florentin et al., 2017). In *P. falciparum*, the nuclear encoded apicoplast ClpC and ClpP protease complex members are essential for apicoplast biogenesis, and conditional mutants of these proteins resulted in growth arrest and apicoplast loss (Florentin et al., 2017, Florentin et al., 2020). Indeed, defects in apicoplast biogenesis were observed in *T. gondii* tachyzoites treated with DNA gyrase inhibitor ciprofloxacin and apicoplast protein translation inhibitor clindamycin (Amber-Johnson and Yeh, 2019). In this study we observed that after 2 days of ATc treatment, apicoplast loss occurs and is partially concomitant with genome loss, which makes it difficult to determine the real direct impact of Prex and DNA gyrase in apicoplast genome replication and how this impacts the organelle function. The evaluation of genome loss in intact apicoplasts for short periods of hours is limited by the slower response of the Tet-inducible system, which prevents investigation of the real impact of the immediate depletion of Prex and DNA gyrase on apicoplast genome replication and transcription.

In conclusion, in this work we demonstrated an essential role for Prex and DNA gyrase in apicoplast maintenance. We also showed that apicoplast genome partitioning occurs in coordination with the parasite cell cycle, but initiates prior to nuclear mitosis and parasite cytokinesis, which ensures that upon apicoplast fission, all daughter cells receive a functional organelle with a genome.

## Appendix A Supplementary data

Supplementary data 1

Supplementary data 2 **Supplementary Fig. S1.** Cryo-immuno electron microscopy of *Toxoplasma gondii* parasites expressing endogenous hemagglutinin (HA) -tagged single strand binding protein (SSB-HA). (A and B) Two different parasites, showing apicoplast localization for SSB (arrowheads). Bars = 200 nm. A, apicoplast; M, mitochondrion; N, nucleus. **Supplementary Fig. S2**. Fluorescence intensity distribution analyses of merge images of *Toxoplasma gondii* apicoplast DNA and Gyrase A (GyrA) shown in Fig. 2A in the main text. (A–F) hemagglutinin (HA)-tagged GyrA (Gyr-HA) parasites were labeled with DAPI for apicoplast and nucleus DNA, anti-Cpn60 for apicoplast lumen and anti-HA for GyrA. The histograms show the distribution of fluorescence signals over the drawn lines at the apicoplast regions in the merge images. For each signal (graph color matches image color) the Y-axis shows fluorescence intensity in pixels and the X-axis shows the distances of signals according to the drawn line. **Supplementary Fig. S3**. Fluorescence intensity distribution analyses of merged images of Gyrase B-myc and GyrA-HA (hemagglutinin) shown in Fig. 2C in the main text. (A–F) Parasites were labeled with anti-myc for GyrB and anti-HA for GyrA. The graphs show the distribution of fluorescence signals over the drawn lines at the apicoplast regions in the merges images. For each signal (graph color matches image color) the Y-axis shows fluorescence intensity in pixels and the X-axis shows the distances of signals according to the drawn lines. **Supplementary Fig. S4**. Super-resolution Structured Illumination Microscopy analysis of *Toxoplasma gondii* parasites expressing endogenous single strand binding protein -hemagglutinin (SSB-HA). (A) Amplification of image shown in Fig. 3A in the main text. White arrowheads point to SSB localization (B) Analysis of SSB-HA parasites after labeling with anti-HA for SSB-HA (green), anti-Cpn60 for apicoplast lumen (red) and the protein ISP1 for parasite cap (blue). **Supplementary Fig. S5**. Fluorescence intensity distribution analyses of merged images of *Toxoplasma gondii* Prex-hemagglutinin (Prex-HA) and apicoplast DNA shown in Fig. 3B in the main text. (A–C) Parasites were labeled with DAPI for apicoplast and nucleus DNA, anti-HA for Prex. The graphs show the distribution of fluorescence signals over the drawn lines across the apicoplast nucleoid in the merged images. For each signal (graph color matches image color) the Y-axis shows fluorescence intensity in pixels and the X-axis shows distances of signals according to the drawn lines. Supplementary Fig. S6. Fluorescence intensity distribution analyses of merged images of *Toxoplasma gondii* hemagglutinin-tagged (HA)-Prex (multi-functional polypeptide containing DNA primase, DNA helicase and DNA polymerase) and single strand binding protein (SSB)-myc shown in Fig. 3C in the main text. (A–F) Parasites were labeled with anti-Cpn60 for apicoplast lumen (blue), anti-HA for Prex (red) and anti-myc for SSB (green). Additionally, parasites were labeled with anti-ISP1 for depiction of mother and daughter cell caps (green). The graphs show the distribution of fluorescence signals over the drawn lines across the Prex and SSB regions in the merged images. For each signal (graph color matches image color) the Y-axis shows fluorescence intensity in pixels and the X-axis shows distances of signals according to the drawn lines. **Supplementary Fig. S7**. Analysis of *Toxoplasma gondii* apicoplast loss by Prex (multi-functional polypeptide containing DNA primase, DNA helicase and DNA polymerase), Gyrase A (GyrA) and GyrB mutants after anhydrotetracycline (ATc) treatment. (A–D) Parental line and mutants of Prex, GyrA and GyrB were grown with ATc for 2, 3 and 6 days, then apicoplast loss was evaluated by counting the immunofluorescence signal obtained via staining with anti-Cpn60 antibody in 100 parasites for each time point. **Supplementary Fig. S8**. *Toxoplasma gondii* apicoplast analysis in Gyrase A (GyrA) mutants by Super-resolution Structured Illumination Microscopy after treatment with anhydrotetracycline (ATc) for 2 days. (A) Non-induced GyrA mutant parasites harboring normal sized apicoplasts. (B) After ATc treatment for 2 days some parasites harbored small sized organelles (arrowheads). Parasites were labeled with anti-Cpn60 for apicoplast (red) and DAPI for DNA (blue). Bars = 1 µm.
