# Supplementary material for: Replication and partitioning of the apicoplast genome of Toxoplasma gondii is linked to the cell cycle and requires DNA polymerase and gyrase
Source: Int J Parasitol. 2021 May;51(6):493–504. doi: 10.1016/j.ijpara.2020.11.004 (PMC8113025; doi:10.1016/j.ijpara.2020.11.004)
Supplement: Supplementary data 1 [file mmc1.docx]

**Supplementary Table S1.** Primers used in this work.

| **Description** | | **Strand** | **Sequence (5’to 3’)** |
| --- | --- | --- | --- |
| 1 | Prex 3’tagging | sense | TACTTCCAATCCAATTTAATGCACCACAATAATGCT GGTCCACGG |
| 2 | Prex 3’tagging | antisense | TCCTCCACTTCCAATTTTAGCCGGCTTGTCTGCCCA GCTGTC |
| 3 | SSB 3’ tagging | sense | TACTTCCAATCCAATTTAATGCACCGTTTTCTGC CCAACTCTG |
| 4 | SSB 3’ tagging | antisense | TCCTCCACTTCCAATTTTAGCGCGGAAAAGGCGATACATGC |
| 5 | GyrA 3’ tagging | sense | TACTTCCAATCCAATTTAATGCCGACGGAAGAAAGGGATA TCG |
| 6 | GyrA 3’ tagging | antisense | TCCTCCACTTCCAATTTTAGCAACTCTAAACAACCAGTCTATCTTC TTG |
| 7 | GyrB cloning into pDT7S4-myc | sense | AGATCTAAAATGAAGGCCTCCTCAGAG |
| 8 | GyrB cloning into pDT7S4-myc | antisense | CCTAGGAACATCCAGCTCTTCCAC |
| 9 | SSB cloning into pDT7S4-myc | sense | GCTAAGATCTATGATTGAACAAGGGTC |
| 10 | SSB cloning into pDT7S4-myc | antisense | GCTACCTAGGGCGGAAAAGGCGATACATG |
| 11 | Prex promoter replacement | sense | GACGAGCCGGGAGACGCGACGACACGAGGCGCCTGAACTTGCGTCGCACTGAATGGTAACCGACAAAACGCGTTC |
| 12 | Prex promoter replacement | antisense | TCACCATCCATTTCTCCACACTTCTTCCGGTACTCAACCGGACGCATAGATCTGGTTGAAGACAGACGAAAGC |
| 13 | GyrA promoter replacement | sense | CGTCCATACGCCAGTTCGTAGTCACGATGCTTGCGTCCCTTTGCCCTCGAGAATGGTAACCGACAAACGCGTTC |
| 14 | GyrA promoter replacement | antisense | AGAGGAAGCAGGAAGCAGACGAGAGGGGAGAGTGAAACACCCCCATAGATCTGGTTGAAGACAGACGAAAGC |
| 15 | SSB promoter insertion | sense | TGAGAGGGCGGCTTTCAGTGCGCCGTGTACAGACGTCCGATATATTTTGCGAATGGTAACCGACAAACGCGTTC |
| 16 | SSB promoter insertion | antisense | TTCCTCCACATATCCTCCTGCGACGAAGAAAAAGACCCTTGTTCAATCATAGATCTGGTTGAAGACAGACGAAAGC |
| 17 | GyrB promoter insertion | sense | AGATCTAAAATGAAGGCCTCCTCAGAG |
| 18 | GyrB promoter insertion | antisense | CCTAGGGAAAGCGATGGAGACAGAG |
| 19 | Native Promoter of GyrB up stream at the translation site in parental and inserted parasites | sense | TGTCTGCAGCTTCTTCGAGTAG |
| 20 | Verification of Native Promoter of GyrB up stream at the translation site or PI | antisense | TCATGCCCTGCGTTTCGCTC |
| 21 | GyrB expression analysis | sense | CGTCTTCACCTCCAGTTCCATC |
| 22 | GyrB expression analysis | antisense | GTGAAGACTGTCTCTAACGC |
| 23 | Sag1 expression control | sense | AACATTGAGCTCCTTGATTCCTG |
| 24 | Sag1 expression control | antisense | GGAACAGTACTGATTGTTGTCTTG |
| 25 | Native Promoter of SSB up stream at the translation site | sense | TCCCAGCAATGGAGACGTC |
| 26 | SSB Native Promoter up stream at the translation site or PI | antisense | AGGTACGCAGAACTCCACTG |
| 27 | GyrA Native Promoter presence in parental and PR parasites | sense | AGTTCGTAGTCACGATGCTTG |
| 28 | GyrAPR confirmation | antisense | ACAGGTGATACTGAGAAG |
| 29 | PrexPR confirmation | sense | TCGGAGCACTTCCCAGTGGCGATC |
| 30 | Verification of native Promoter of Prex in parental and PR parasites | sense | CTCCTCCACAGTAAATGTGCAT |
| 31 | Verification of native Promoter of Prex in parental and PR parasites | antisense | GTTTCGCGTCCGCAACAGGATGTGT |
| 32 | T7S4 promoter/DHFR replacement/insertion confirmation #1 | sense | CGTTTCCTCTTCCCTCAAA |
| 33 | T7S4 promoter/DHFR replacement #2 | antisense | CGCACGGCAGTCAGATAACAGGTGTA |

Prex, multi-functional polypeptide containing DNA primase, DNA helicase and a C-terminal domain containing both 3’5’-exonuclease and DNA polymerase; Gyr, gyrase; SSB, single strand binding protein; DHFR,dihydrofolate reductase; PR, promoter replacement; PI, promoter insertion; pDT7S4-myc, plasmid containing the pyrimethamine-resistant DHFR gene, promoter T7S4 and myc tag.

**Supplementary** **Table S2.** Statistical analysis^a^ of nucleoid types within stages of the experiment shown in Fig. 5 in the main text.

| Stage | One round nucleoid vs. one dumbbell | One round nucleoid vs.  two nucleoids | One dumbbell vs.  two nucleoids |
| --- | --- | --- | --- |
| 1 | *P* < 0.001 | *P* < 0.001 | Ns |
| 2 | Ns^b^ | *P* < 0.01 | *P* < 0.05 |
| 3 | Ns | Ns | Ns |
| 4 | *P* < 0.01 | *P* < 0.0001 | Ns |
| 5 | Ns | *P* < 0.0001 | *P* < 0.0001 |
| 6 | Ns | *P* < 0.0001 | *P* < 0.0001 |

^a^ Two-way ANOVA with Tukey’s multiple comparisons test within each row/compare columns

^b^ Ns – Not significant

**Supplementary Table S3.** Statistical analysis^a^ of nucleoid types between stages of the experiment shown in Fig. 5 in the main text.

| Stage | One round nucleoid | One dumbbell | Two nucleoids |
| --- | --- | --- | --- |
| 1 vs. 2 | Ns^b^ | Ns | Ns |
| 1 vs. 3 | *P* < 0.001 | Ns | *P* < 0.05 |
| 1 vs. 4 | *P* < 0.0001 | Ns | *P* < 0.001 |
| 1 vs. 5 | *P* < 0.0001 | Ns | *P* < 0.0001 |
| 1 vs. 6 | *P* < 0.0001 | Ns | *P* < 0.0001 |
| 2 vs. 3 | *P* < 0.05 | Ns | Ns |
| 2 vs. 4 | *P* < 0.01 | Ns | *P* < 0.001 |
| 2 vs. 5 | *P* < 0.001 | Ns | *P* < 0.0001 |
| 2 vs. 6 | *P* < 0.001 | *P* < 0.01 | *P* < 0.0001 |
| 3 vs. 4 | Ns | Ns | Ns |
| 3 vs. 5 | Ns | Ns | *P* < 0.001 |
| 3 vs. 6 | Ns | *P* < 0.01 | *P* < 0.0001 |
| 4 vs. 5 | Ns | Ns | Ns |
| 4 vs. 6 | Ns | *P* < 0.01 | *P* < 0.001 |
| 5 vs. 6 | Ns | Ns | Ns |

^a^ Two-way ANOVA with Tukey’s multiple comparisons test within each column/compare rows

^b^ Ns – Not significant
