# Supplementary material for: Replication and partitioning of the apicoplast genome of Toxoplasma gondii is linked to the cell cycle and requires DNA polymerase and gyrase
Source: Int J Parasitol. 2021 May;51(6):493–504. doi: 10.1016/j.ijpara.2020.11.004 (PMC8113025; doi:10.1016/j.ijpara.2020.11.004)

Supplementary Figures

Supplementary Fig. S1

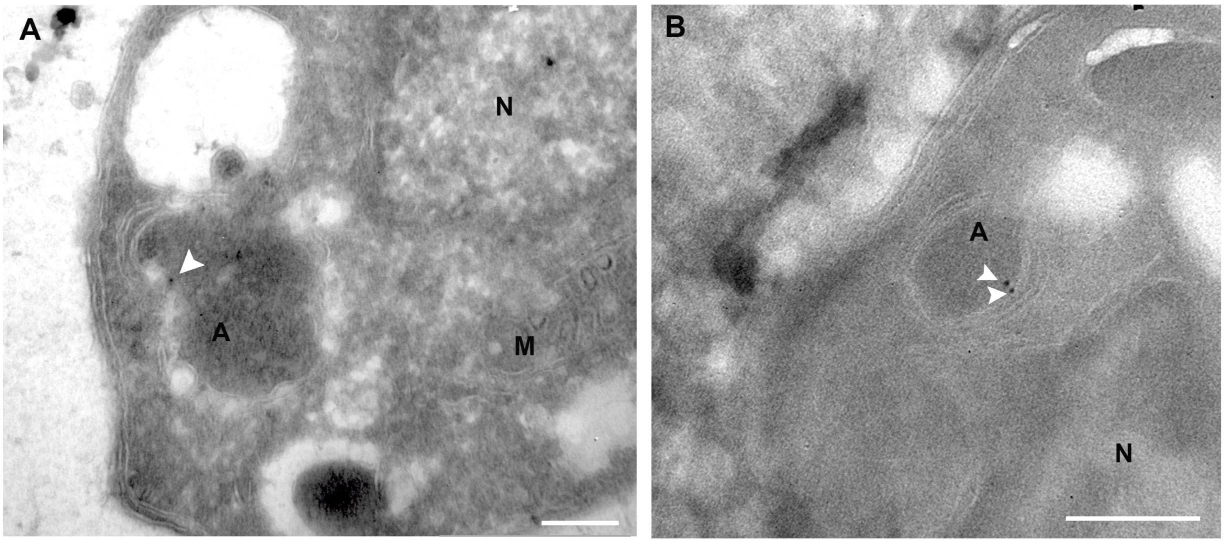

Supplementary Fig. S2

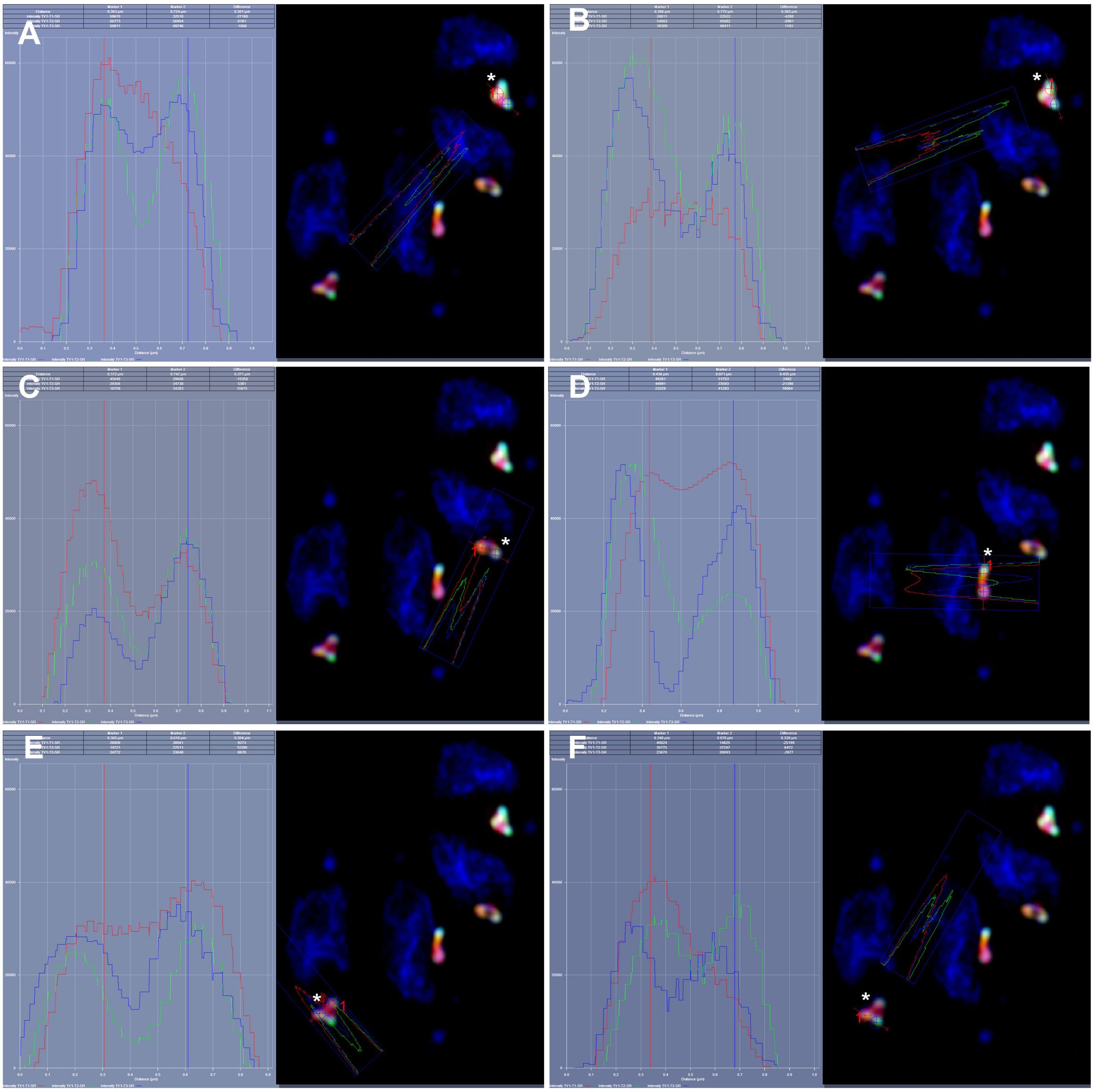

Supplementary Fig. S3

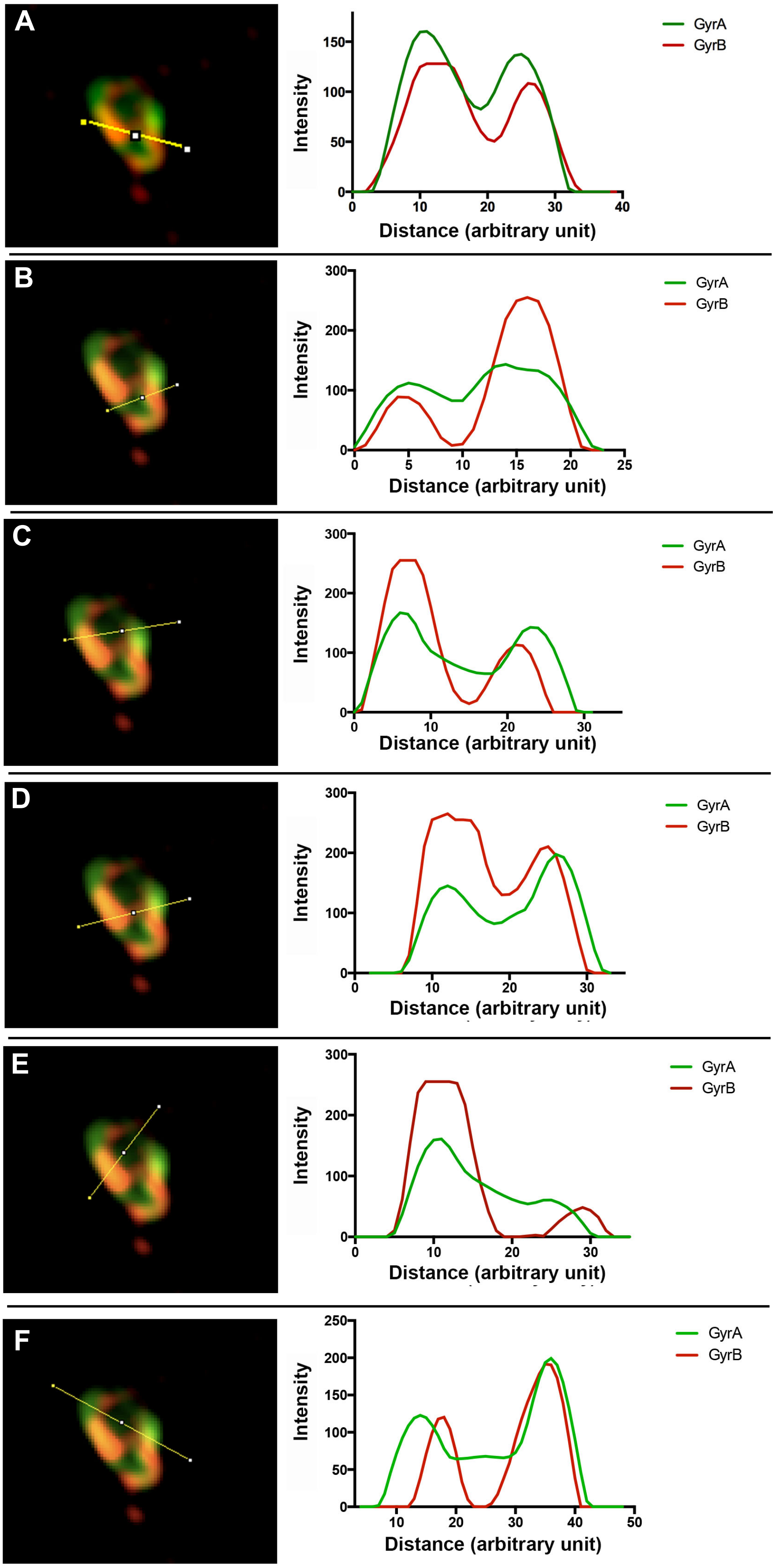

Supplementary Fig. S4

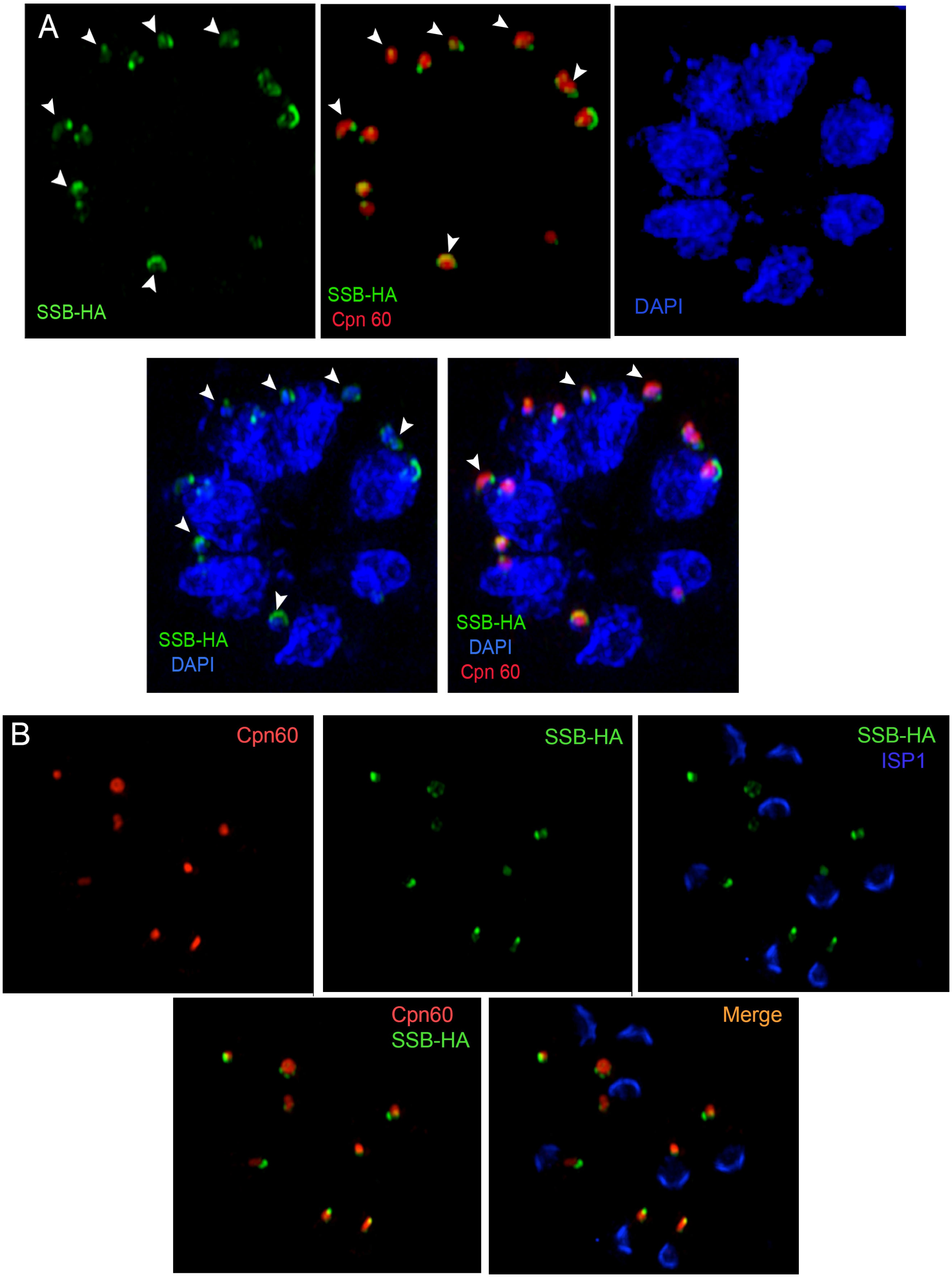

Supplementary Fig. S5

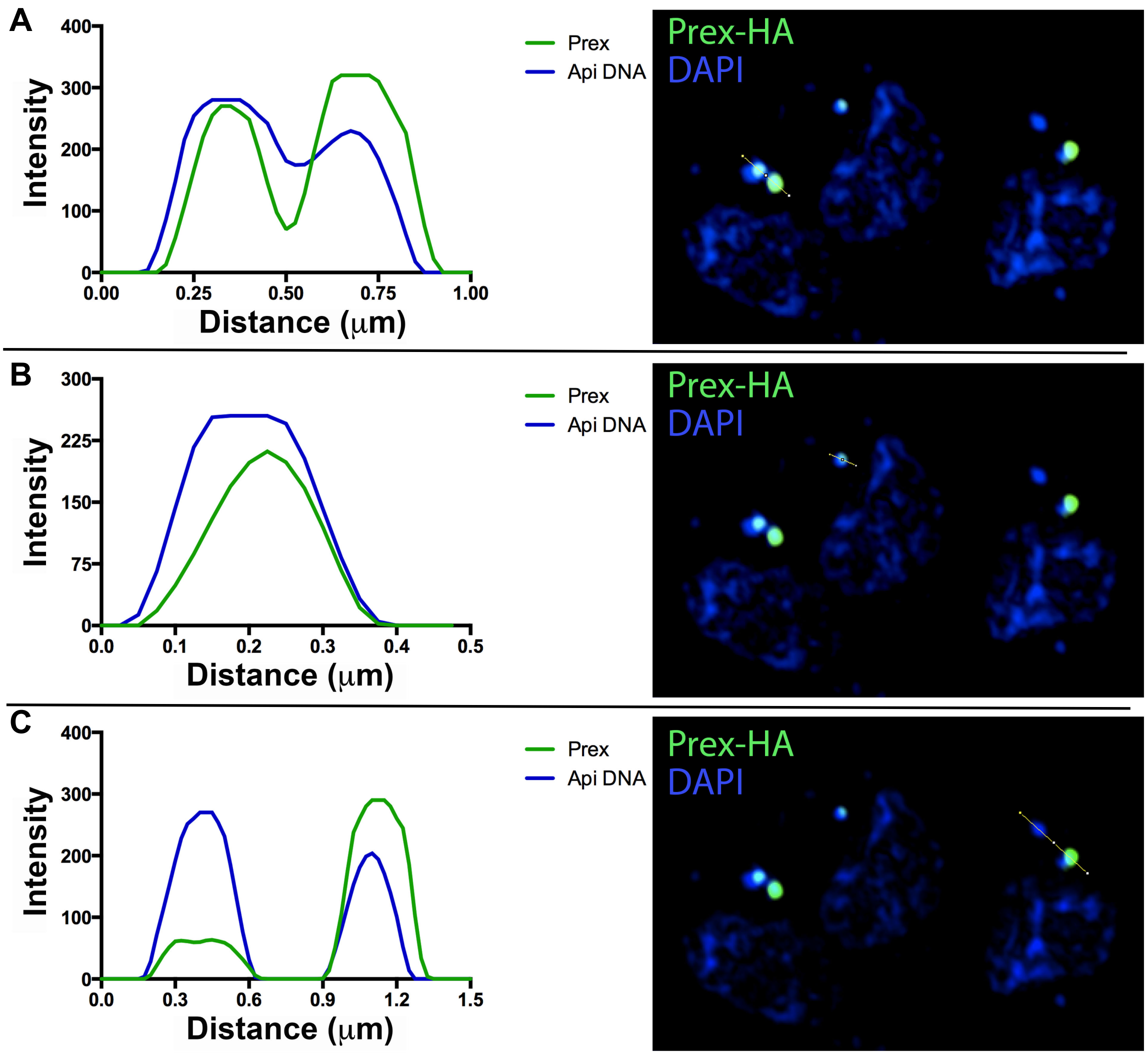

Supplementary Fig. S6

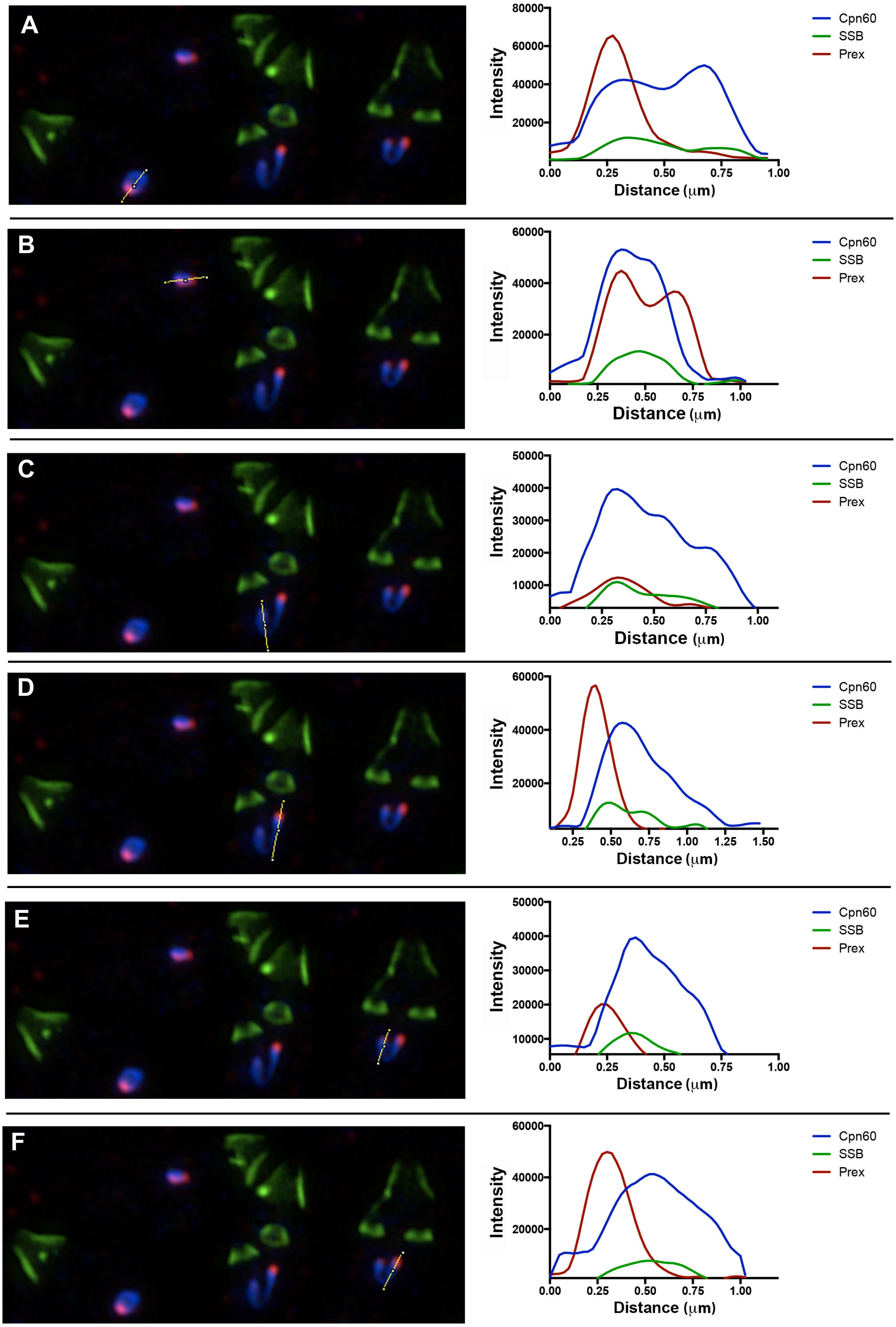

Supplementary Fig. S7

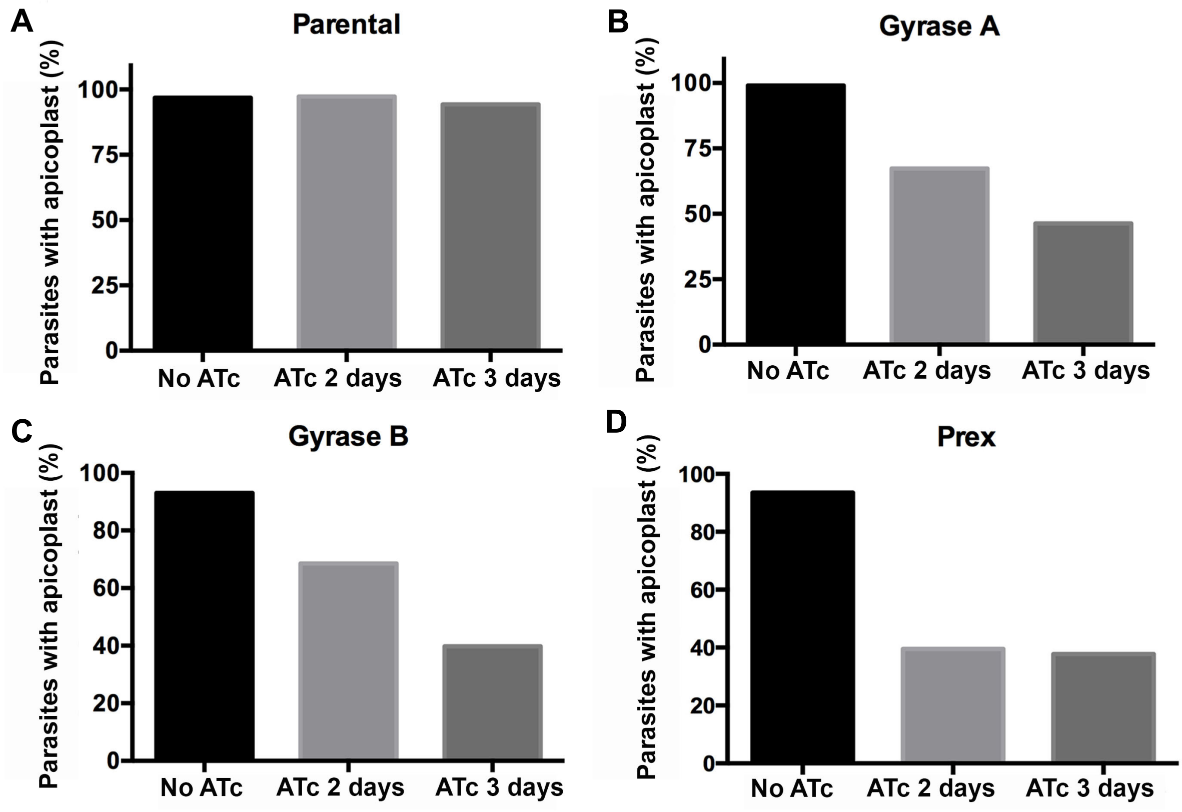

Supplementary Fig. S8

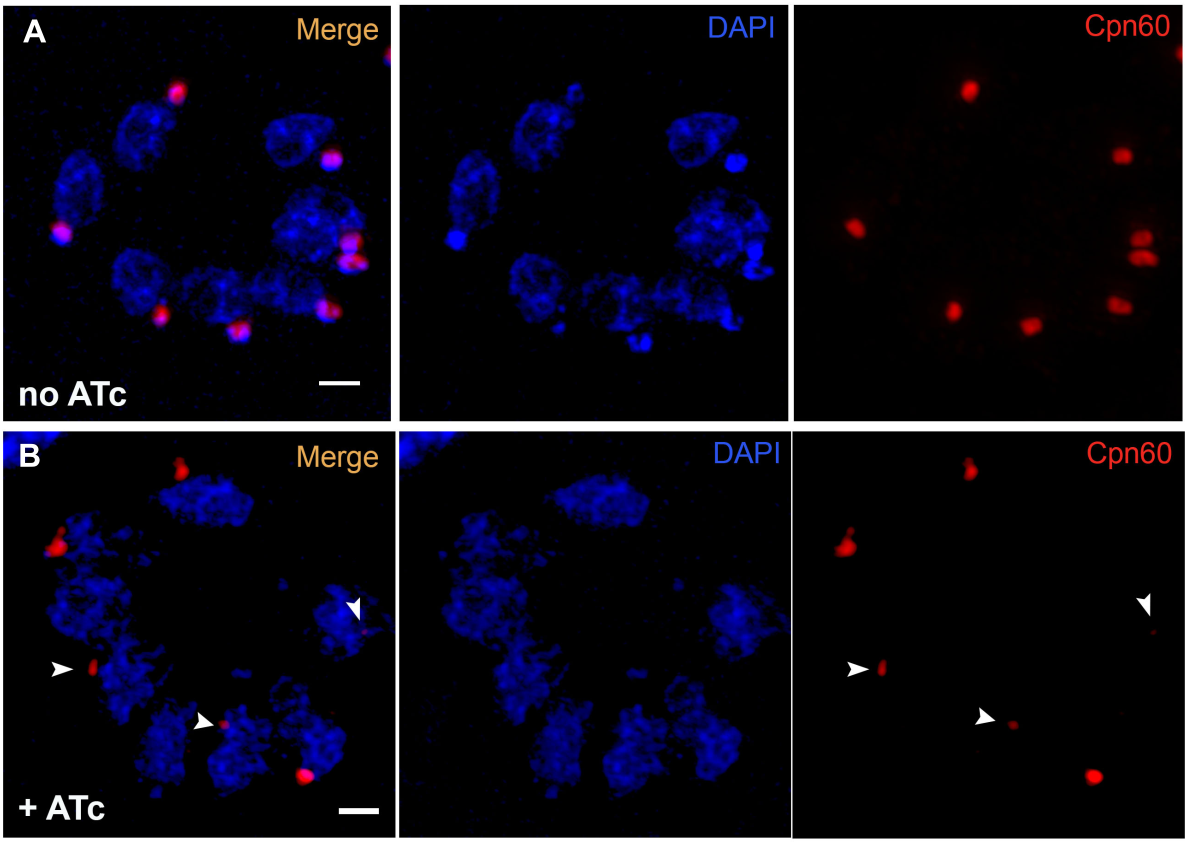

Supplement: Supplementary data 2 — Supplementary Fig. S1. Cryo-immuno electron microscopy of Toxoplasma gondii parasites expressing endogenous hemagglutinin (HA) -tagged single strand binding protein (SSB-HA). (A and B) Two different parasites, showing apicoplast localization for SSB (arrowheads). Bars = 200 nm. A, apicoplast; M, mitochondrion; N, nucleus. Supplementary Fig. S2. Fluorescence intensity distribution analyses of merge images of Toxoplasma gondii apicoplast DNA and Gyrase A (GyrA) shown in Fig. 2A in the main text. (A–F) hemagglutinin (HA)-tagged GyrA (Gyr-HA) parasites were labeled with DAPI for apicoplast and nucleus DNA, anti-Cpn60 for apicoplast lumen and anti-HA for GyrA. The histograms show the distribution of fluorescence signals over the drawn lines at the apicoplast regions in the merge images. For each signal (graph color matches image color) the Y-axis shows fluorescence intensity in pixels and the X-axis shows the distances of signals according to the drawn line. Supplementary Fig. S3. Fluorescence intensity distribution analyses of merged images of Gyrase B-myc and GyrA-HA (hemagglutinin) shown in Fig. 2C in the main text. (A–F) Parasites were labeled with anti-myc for GyrB and anti-HA for GyrA. The graphs show the distribution of fluorescence signals over the drawn lines at the apicoplast regions in the merges images. For each signal (graph color matches image color) the Y-axis shows fluorescence intensity in pixels and the X-axis shows the distances of signals according to the drawn lines. Supplementary Fig. S4. Super-resolution Structured Illumination Microscopy analysis of Toxoplasma gondii parasites expressing endogenous single strand binding protein -hemagglutinin (SSB-HA). (A) Amplification of image shown in Fig. 3A in the main text. White arrowheads point to SSB localization (B) Analysis of SSB-HA parasites after labeling with anti-HA for SSB-HA (green), anti-Cpn60 for apicoplast lumen (red) and the protein ISP1 for parasite cap (blue). Supplementary Fig. S5. Fluores [file mmc2.pdf]
